# Behavioral pathways linking influenza vaccine knowledge, uptake, vaccination intention, and recommendation behavior among healthcare workers: a cross-sectional study in Shijingshan District, Beijing

**DOI:** 10.3389/fpubh.2026.1886295

**Published:** 2026-09-09

**Authors:** Hongbo Shi, Xiao Zhao, Lin Zhou, Lingyu Zhang, Wei Qin, Junli Lian, Yanhui Li, Jin Wu

**Affiliations:** Shijingshan District Center for Disease Control and Prevention, Beijing, China

**Keywords:** healthcare workers, influenza vaccination, observed-variable path analysis, recommendation behavior, vaccination intention, vaccination uptake

## Abstract

**Objective:**

Healthcare workers are a priority group for influenza vaccination and play an important role in recommending vaccination to patients. However, the relationships among influenza vaccine knowledge, vaccination uptake, future vaccination intention, and recommendation behavior remain insufficiently understood. This study aimed to examine these associations and their behavioral pathway relationships among healthcare workers in Shijingshan District, Beijing.

**Methods:**

A cross-sectional survey was conducted in January 2026 among healthcare workers from medical institutions in Shijingshan District, Beijing. Of 4,366 respondents, 4,238 valid questionnaires were included in the final analysis. Data were collected on influenza vaccine-related knowledge, vaccination uptake during the current influenza season, intention to receive influenza vaccination in the next influenza season, and recommendation behavior toward patients. Logistic regression analyses were used to identify associated factors, and SEM-based path analysis was performed to examine the direct and indirect associations among influenza vaccine-related knowledge, vaccination uptake, future vaccination intention, and recommendation behavior.

**Results:**

Among the 4,238 participants, 22.1% received influenza vaccination during the current influenza season, 43.7% intended to be vaccinated in the next influenza season, and 82.0% reported recommending influenza vaccination to patients. Logistic regression analyses showed that knowledge score, training/publicity, free vaccination policy, institutional support, and prior vaccination were significantly associated with vaccination uptake, future vaccination intention, and recommendation behavior. The SEM-based path analysis model showed a good fit (CFI = 0.957, TLI = 0.941, RMSEA = 0.041, SRMR = 0.036). Knowledge score was positively associated with vaccination uptake (*β* = 0.09, *p* < 0.001). Current-season vaccination uptake strongly predicted future vaccination intention (*β* = 0.47, *p* < 0.001), and future vaccination intention was the strongest direct predictor of recommendation behavior (*β* = 0.41, *p* < 0.001). Mediation analysis showed that knowledge score indirectly influenced recommendation behavior through current-season vaccination uptake and future vaccination intention.

**Conclusion:**

Influenza vaccine-related behaviors among healthcare workers followed a progressive behavioral pathway from knowledge to vaccination uptake, then to future vaccination intention, and finally to recommendation behavior. Interventions targeting knowledge, institutional support, and actual vaccination uptake may provide evidence for developing targeted interventions among healthcare workers in similar settings.

## 1 Introduction

Influenza remains a major public health concern worldwide because of its substantial morbidity, mortality, and economic burden. The World Health Organization has estimated that seasonal influenza causes more than 1 billion infections annually and 290,000–650,000 respiratory deaths worldwide (1). Healthcare workers are recognized as a priority group for influenza vaccination, not only because of their increased occupational exposure to respiratory pathogens, but also because they may transmit influenza to vulnerable patients within healthcare settings (2). In addition, healthcare workers serve as important sources of vaccine information and recommendations for patients, making their own vaccination-related attitudes and behaviors highly relevant to broader vaccination promotion efforts (3).

Despite clear recommendations, influenza vaccination coverage remains suboptimal in many populations. Previous studies in China have shown persistently low influenza vaccination uptake in priority groups, even where vaccination is strongly encouraged (4). Existing evidence suggests that vaccination behavior is influenced by multiple factors, including vaccine-related knowledge, training and publicity, service accessibility, financial support, and organizational climate. In particular, institutional support, such as free vaccination policies, on-site vaccination services, and positive workplace attitudes, may substantially shape actual uptake among healthcare workers (5). However, vaccination is not merely a single behavior; it is part of a broader decision-making process that may extend from knowledge acquisition to behavioral adoption and, ultimately, to professional recommendation practices (6).

Most previous studies have focused on a single outcome, such as vaccination uptake or vaccination willingness, and have often relied on conventional regression approaches to identify associated factors (7). While such studies are valuable, they are limited in their ability to explain how knowledge and contextual support may jointly influence subsequent behaviors. In healthcare workers, actual vaccination uptake, future vaccination intention, and recommendation behavior toward patients may represent different but sequential stages of vaccine-related behavior (8). Understanding these stages together is important because healthcare workers who accept vaccination themselves may be more likely to maintain future vaccination intention and, in turn, recommend influenza vaccination to patients. Thus, exploring the behavioral pathway from knowledge to uptake, intention, and recommendation may provide a more comprehensive understanding of influenza vaccination-related behaviors in this population (9).

Shijingshan District in Beijing provides a relevant setting for examining this issue, given its urban healthcare system and the important role of frontline and public health personnel in influenza prevention and control. In this study, we conducted a cross-sectional survey among healthcare workers in medical institutions in Shijingshan District, Beijing. We aimed to identify factors associated with influenza vaccination uptake during the current influenza season, intention to receive vaccination in the next influenza season, and recommendation behavior toward patients. Furthermore, we sought to examine the behavioral pathway relationships among influenza vaccine knowledge, vaccination uptake, future vaccination intention, and recommendation behavior, while also considering the potential influence of training/publicity and institutional support factors. We intended to provide evidence for targeted interventions to improve influenza vaccination-related behaviors among healthcare workers.

## 2 Methods

### 2.1 Study design and participants

This study employed a cross-sectional survey design and was conducted in January 2026 among healthcare workers engaged in medical-related work in registered medical institutions in Shijingshan District, Beijing. A stratified two-stage sampling method was adopted. In the first stage, medical institutions in the urban area were stratified by institutional level, and 72 medical institutions were randomly selected, including 4 tertiary institutions, 2 secondary institutions, 6 primary institutions, and 60 ungraded institutions (e.g., traditional Chinese medicine clinics, dental clinics, general outpatient departments, and medical cosmetology clinics). In the second stage, within each selected institution, participants were randomly sampled in proportion to the distribution of occupational posts, including administrative staff (e.g., hospital administration office and medical affairs department), public health staff (e.g., public health department, infection control department, and health education department), medical technical staff (e.g., laboratory and imaging departments), and clinical staff (e.g., internal medicine, surgery, obstetrics and gynecology, and pediatrics). All participants voluntarily completed the questionnaire after providing informed consent.

Eligible participants were healthcare workers who (1) were aged 18 years or older; (2) were registered in medical institutions in Shijingshan District and engaged in medical-related work; (3) were able to complete the questionnaire independently; and (4) voluntarily agreed to participate in the survey and provided informed consent. Individuals were excluded if they (1) were away from work for further training or external placement during the survey period, or (2) had possible contraindications to influenza vaccination.

### 2.2 Sample size calculation

The sample size was estimated using the standard formula for cross-sectional studies:

$$n={Z^{2}}_{1-\alpha /2}\times p(1-p)/d^{2}$$

where *n* is the required sample size, *p* is the expected proportion, *d* is the allowable error, and *α* is the significance level. With a significance level of 0.05 (*Z*_1 − *α*/2_ = 1.96), the expected proportion of influenza vaccination willingness was set at 0.60 based on previous studies (10), and the allowable error was set at 0.05. The calculated minimum sample size was 369. After accounting for an anticipated questionnaire response rate of 85% and a valid response rate of 80%, the required minimum sample size was estimated to be 543. The final sample size included in this study was 4,238, which exceeded the minimum requirement.

### 2.3 Questionnaire and measures

#### 2.3.1 Questionnaire

The questionnaire used in this study was adapted from the Survey Questionnaire on Influenza Vaccination Uptake and Recommendation Intention Among General Practitioners developed by Professor Lvzhao Feng’s team at Peking Union Medical College (10). Additional items applicable to healthcare workers in Shijingshan District, Beijing, were incorporated into the original questionnaire. This instrument has been widely used to assess influenza vaccination uptake and recommendation intention among healthcare professionals. The original questionnaire demonstrated good reliability and validity, with an intraclass correlation coefficient (ICC) of 0.83 for test–retest reliability and a Cronbach’s alpha coefficient of 0.72 for internal consistency. Factor analysis showed that the factor loadings of all dimensions were greater than 0.5, indicating acceptable construct validity (10). In the present study, the original questionnaire was retained as the primary instrument, and only a limited number of additional items related to institutional vaccination support, vaccination service availability, and workplace vaccination environment were added to reflect the local healthcare context in Shijingshan District. Before the formal survey, a pilot study was conducted among 150 healthcare workers who were not included in the final analysis. The adapted questionnaire showed satisfactory reliability, with an ICC of 0.86 and a Cronbach’s alpha coefficient of 0.78. Exploratory factor analysis demonstrated acceptable construct validity, with all retained items showing factor loadings greater than 0.50 (range: 0.56–0.84). These results supported the reliability and applicability of the questionnaire for the present study.

The questionnaire consisted of six sections.

1. Questionnaire instructions and informed consent: Before completing the questionnaire, participants were informed of the main purpose and significance of the study, as well as the specific requirements for questionnaire completion. Informed consent was obtained from all participants before the survey.
2. Sociodemographic characteristics: This section collected information on sex, age, highest educational attainment, occupation, years of professional practice, type of employing institution, occupational post, professional title, monthly income, history of influenza-like illness (ILI) symptoms, and participation in influenza vaccine-related training or publicity activities.
3. Influenza vaccine-related knowledge: This section included six knowledge items. Three were true-or-false questions, two were single-choice questions, and one was a multiple-choice question. Correct responses were scored as 1 and incorrect responses as 0. For the multiple-choice item, each option was scored separately as 1 for a correct response and 0 for an incorrect response. The total knowledge score was calculated by summing the scores of all items, with a possible range of 0–10; a higher score indicated a higher level of influenza vaccine-related knowledge.
4. Current influenza vaccination status: This section assessed healthcare workers’ influenza vaccination uptake during the 2025–2026 influenza season, as well as institutional vaccination support, including whether the employing institution provided influenza vaccination services, whether a free influenza vaccination policy was available, and the institution’s attitude toward influenza vaccination among healthcare workers.
5. Intention to receive influenza vaccination: This section assessed healthcare workers’ intention to receive influenza vaccination during the 2026–2027 influenza season. Drivers and barriers to vaccination intention were further explored through three dimensions: thoughts and feelings, social support, and practical issues.
6. Recommendation intention and attitudes toward the vaccine prescription model: This section investigated healthcare workers’ willingness to recommend influenza vaccination to patients, reasons for unwillingness to recommend vaccination, and their attitudes toward the vaccine prescription model.

The variables derived from the questionnaire and their coding schemes used in the analyses are described below.

#### 2.3.2 Variable definitions

In the main analyses, the influenza vaccine-related knowledge score was treated as a continuous variable, with higher scores indicating a higher level of knowledge.

Three core outcome variables were defined. Vaccination uptake during the current influenza season was coded as 1 for “yes” and 0 for “no.” Intention to receive influenza vaccination in the next influenza season was dichotomized as 1 for “willing” and 0 for “unwilling” or “undecided.” Willingness to recommend influenza vaccination to patients was coded as 1 for “willing” and 0 for “unwilling.”

Organizational support-related variables included participation in influenza vaccine-related training or publicity activities (yes = 1, no = 0), provision of influenza vaccination services by the employing institution (yes = 1, no = 0), and availability of a free vaccination policy (yes = 1, no = 0). Institutional attitude toward influenza vaccination among healthcare workers was entered into the regression models as a categorical variable with three levels: requiring vaccination, encouraging vaccination without requiring it, and neither requiring nor encouraging vaccination.

### 2.4 Quality control

Several measures were implemented to ensure the quality of the survey.

#### 2.4.1 Preparatory phase

Before the formal survey, a pilot test was conducted among colleagues at the investigators’ institution who were not included in the final study sample. Feedback from these healthcare workers was used to evaluate the performance of the questionnaire in practice, identify potential problems in questionnaire design, and make necessary revisions to further optimize questionnaire quality.

#### 2.4.2 Data management

The research team regularly reviewed and organized the collected data through the Wenjuanxing platform. Data were carefully checked and cleaned to ensure accuracy and reliability. When logical inconsistencies were identified, the participants were contacted in a timely manner for verification and correction.

#### 2.4.3 Logical validation

Several procedures were applied to improve data validity. First, the questionnaire was completed online, with skip logic embedded where necessary, and participants were required to complete all relevant items before submission. Second, restrictions on answering frequency and completion time were set; questionnaires completed in less than 150 s were considered invalid, and each participant was allowed to respond only once. Duplicate responses were excluded, and fraudulent behaviors such as completing the questionnaire on behalf of others were prohibited. Third, a quality-control item based on basic medical knowledge (“Which of the following indicators belongs to a patient’s vital signs?”) was included to identify inattentive respondents. This item could be answered correctly by healthcare workers with normal attention and basic professional knowledge, thereby helping to improve data validity.

#### 2.4.4 Project implementation

A unified questionnaire was designed and distributed through the Wenjuanxing platform. The questionnaire notice, together with a QR code for access, was issued through the Shijingshan District Health Commission. In each participating medical institution, disease control supervisors were designated as survey coordinators. These coordinators were required to be conscientious and detail-oriented and received standardized training before the survey. During data collection, they provided timely support to participants through WeChat groups and reported any problems encountered in questionnaire completion to the research team. Before the survey was launched, the disease control departments of the participating medical institutions further disseminated the survey notice and mobilized eligible staff to complete the questionnaire.

### 2.5 Statistical analysis

All statistical analyses were conducted using R version 4.4.1. Descriptive analyses were first performed for all study variables. Categorical variables were presented as frequencies and percentages. Continuous variables were described as means ± standard deviations if normally distributed, or as medians (P25, P75) if skewed.

Univariate logistic regression analyses were conducted to examine the associations between candidate variables and each of the three core outcomes, including influenza vaccination uptake during the current influenza season, intention to receive influenza vaccination in the next influenza season, and willingness to recommend influenza vaccination to patients. Variables with statistical significance in the univariate logistic regression analyses, together with variables considered relevant based on professional judgment, were entered into multivariable logistic regression models to identify factors independently associated with each outcome. Crude odds ratios (ORs), adjusted odds ratios (aORs), and 95% confidence intervals (95% CIs) were calculated. Multicollinearity among candidate variables was assessed using variance inflation factors (VIFs), with VIF values <5 considered indicative of acceptable collinearity. Variables with *p* < 0.10 in univariate logistic regression analyses, together with clinically relevant factors identified from previous studies, were included in multivariable logistic regression models. The enter method was used for model construction. Model performance was evaluated using the Hosmer–Lemeshow goodness-of-fit test, Nagelkerke *R*^2^, and the area under the receiver operating characteristic curve (AUC).

To further examine whether the associations with current-season vaccination uptake differed across organizational support contexts, stratified analyses were conducted according to whether a free vaccination policy was available and the institution’s attitude toward influenza vaccination among healthcare workers.

Additional analyses were performed based on the Behavioral and Social Drivers (BeSD) framework to supplement the analysis of vaccination intention. Drivers and barriers to future influenza vaccination intention were explored from the dimensions of thoughts and feelings, social support, and practical issues. In addition, descriptive analyses were conducted for reasons for unwillingness to recommend influenza vaccination to patients and for attitudes toward the vaccine prescription model.

To assess the potential influence of common method bias arising from the use of self-reported questionnaire data, Harman’s single-factor test was performed. All measurement items included in the questionnaire were entered into an exploratory factor analysis without rotation, and the proportion of variance explained by the first factor was examined. A value below 50% was considered indicative of the absence of substantial common method bias.

Structural equation modeling (SEM)-based observed-variable path analysis was further conducted to examine the behavioral pathway relationships among influenza vaccine-related knowledge, current-season vaccination uptake, future vaccination intention, and recommendation behavior. All variables included in the model were directly measured observed variables rather than latent constructs. Therefore, the analysis focused on estimating structural associations among observed indicators, and conventional measurement model assessments (e.g., confirmatory factor analysis, factor loadings, composite reliability, and average variance extracted) were not applicable. Based on the theoretical framework and multivariable regression results, knowledge score, vaccination uptake during the current influenza season, intention to receive influenza vaccination in the next influenza season, and recommendation behavior were included as core behavioral pathway variables, while training/publicity, free vaccination policy, provision of vaccination services, and institutional attitude were included as organizational support-related variables. Model fit was evaluated using the comparative fit index (CFI), Tucker–Lewis index (TLI), root mean square error of approximation (RMSEA), and standardized root mean square residual (SRMR). A two-sided *p* value of <0.05 was considered statistically significant. Although the model was composed of directly measured observed variables rather than latent constructs, an SEM-based observed-variable path analysis framework was adopted because the primary objective was to simultaneously examine a theoretically hypothesized network of direct and indirect associations among multiple behavioral outcomes. Compared with separate regression models, this approach allows the estimation of multiple interconnected behavioral pathways within a unified analytical framework and enables the assessment of indirect associations among knowledge, vaccination uptake, future vaccination intention, and recommendation behavior. Because several endogenous variables were binary outcomes, the model was estimated using the weighted least squares mean and variance adjusted (WLSMV) estimator, which is appropriate for models containing categorical observed variables.

### 2.6 Ethical considerations

This study was approved by the Ethics Committee of the Shijingshan District Center for Disease Control and Prevention, Beijing (approval number: SJSCDC2025021). All participants provided informed consent before completing the questionnaire, and the informed consent statement was presented in the introductory section of the electronic questionnaire. Participation was voluntary, and the survey was conducted anonymously. The confidentiality of all collected information was strictly protected throughout the study.

## 3 Results

### 3.1 Characteristics of the study participants

A total of 4,366 questionnaires were collected, of which 4,238 valid questionnaires were included in the final analysis. The characteristics of the study participants are shown in Table 1. Most participants were female (79.1%), worked in tertiary medical institutions (68.7%), and held clinical posts (57.0%). The median knowledge score was 7 (P25, P75: 6, 8). Overall, 22.1% of participants received influenza vaccination during the 2025–2026 season, 43.7% intended to be vaccinated in the 2026–2027 season, and 82.0% reported willingness to recommend influenza vaccination to patients.

**Table 1:** Sociodemographic, occupational, and influenza vaccination-related characteristics of the study participants (*N* = 4,238).

Characteristic		*n* (%) or median (P25, P75)	
Sex	Male	886	(20.9)
	Female	3,352	(79.1)
Age group, years	18–29	837	(19.7)
	30–39	1,670	(39.4)
	40–49	1,135	(26.8)
	≥50	596	(14.1)
Educational level	Secondary school or below	66	(1.6)
	Junior college	687	(16.2)
	Bachelor’s degree	2,589	(61.1)
	Master’s degree or above	896	(21.1)
Years of professional practice	<5	780	(18.4)
	5–9	717	(16.9)
	10–19	1,647	(38.9)
	≥20	1,094	(25.8)
Type of employing institution	Ungraded medical institution	215	(5.1)
	Primary medical institution	830	(19.6)
	Secondary medical institution	280	(6.6)
	Tertiary medical institution	2,913	(68.7)
Occupational post	Administrative staff	260	(6.1)
	Public health staff	247	(5.8)
	Medical technical staff	1,314	(31.0)
	Clinical staff	2,417	(57.0)
Professional title	No professional title	243	(5.7)
	Junior title	1,616	(38.1)
	Intermediate title	1,842	(43.5)
	Senior title	537	(12.7)
Monthly income	<5,000 CNY	339	(8.0)
	5,000–9,999 CNY	2,417	(57.0)
	≥10,000 CNY	1,234	(29.1)
	Refused to disclose	248	(5.9)
Occupation	Physician	1,507	(35.6)
	Nurse	1,909	(45.0)
	Other healthcare workers	822	(19.4)
History of influenza-like illness symptoms in the past year	Yes	1,218	(28.7)
	No or unsure	3,020	(71.2)
Participation in influenza vaccine-related training/publicity	Yes	3,603	(85.0)
	No	635	(15.0)
Institution provides influenza vaccination services	Yes	3,505	(82.7)
	No	733	(17.3)
Free influenza vaccination policy	Yes	3,695	(87.2)
	No	543	(12.8)
Institutional attitude toward influenza vaccination among healthcare workers	Requiring vaccination	743	(17.5)
	Encouraging without requiring	3,167	(74.7)
	Neither requiring nor encouraging	328	(7.8)
Knowledge score, median (P25, P75)		7	(6, 8)
Received influenza vaccination during the 2025–2026 season	Yes	936	(22.1)
	No	3,302	(77.9)
Intention to receive influenza vaccination in the 2026–2027 season	Willing	1,852	(43.7)
	Unwilling/Undecided	2,386	(56.3)
Willingness to recommend influenza vaccination to patients	Willing	3,474	(82.0)
	Unwilling	764	(18.0)

### 3.2 Factors associated with influenza vaccination uptake during the 2025–2026 season

The univariate and multivariable logistic regression results for influenza vaccination uptake during the 2025–2026 season are presented in Table 2. In the multivariable model, years of professional practice, type of employing institution, occupational post, participation in influenza vaccine-related training/publicity, provision of vaccination services by the employing institution, free vaccination policy, institutional attitude toward influenza vaccination, and knowledge score were independently associated with vaccination uptake. Participation in training/publicity, the availability of a free vaccination policy, a supportive institutional attitude, and higher knowledge score were all positively associated with vaccination uptake. Knowledge score was positively associated with influenza vaccination uptake (aOR = 1.220, 95% CI: 1.161–1.282, *p* < 0.001). Before multivariable logistic regression analysis, multicollinearity among candidate variables was assessed using VIFs, and no significant multicollinearity was identified (all VIF values <3). The final model showed good calibration according to the Hosmer–Lemeshow test (*p* = 0.389) and acceptable discrimination (AUC = 0.812).

**Table 2:** Univariate and multivariable logistic regression analyses of factors associated with influenza vaccination uptake during the 2025–2026 season.

Variable	Category/Unit	Univariate OR (95% CI)	*p* value	Multivariable aOR (95% CI)	*p* value
Sex	Male	Reference	–	Reference	–
	Female	0.942 (0.789–1.124)	0.505	–	–
Age group, years	18–29	Reference	–	Reference	–
	30–39	0.909 (0.746–1.107)	0.343	–	–
	40–49	0.798 (0.643–0.990)	0.041	–	–
	≥50	1.047 (0.819–1.338)	0.713	–	–
Educational level	Secondary school or below	Reference	–	–	–
	Junior college	0.813 (0.464–1.424)	0.470	–	–
	Bachelor’s degree	0.683 (0.397–1.173)	0.167	–	–
	Master’s degree or above	0.652 (0.374–1.138)	0.133	–	–
Years of professional practice	≥20	Reference	–	Reference	–
	<5	1.232 (0.996–1.525)	0.054	1.681 (1.322–2.136)	<0.001
	5–9	0.918 (0.730–1.153)	0.461	1.324 (1.027–1.706)	0.030
	10–19	0.852 (0.707–1.026)	0.090	1.136 (0.923–1.398)	0.230
Type of employing institution	Tertiary medical institution	Reference	–	Reference	–
	Secondary medical institution	1.537 (1.152–2.050)	0.003	1.766 (1.288–2.421)	<0.001
	Primary medical institution	2.759 (2.327–3.271)	<0.001	2.474 (2.030–3.014)	<0.001
	Ungraded medical institution	1.388 (0.995–1.935)	0.054	3.005 (1.984–4.551)	<0.001
Occupational post	Clinical staff	Reference	–	Reference	–
	Administrative staff	1.396 (1.041–1.873)	0.026	1.406 (1.012–1.953)	0.042
	Public health staff	3.379 (2.582–4.423)	<0.001	2.071 (1.516–2.830)	<0.001
	Medical technical staff	0.996 (0.843–1.177)	0.963	0.904 (0.754–1.084)	0.278
Professional title	No professional title	Reference	–	–	–
	Junior title	0.755 (0.556–1.025)	0.072	–	–
	Intermediate title	0.751 (0.554–1.017)	0.064	–	–
	Senior title	0.707 (0.498–1.004)	0.053	–	–
Monthly income	<5,000 CNY	Reference	–	–	–
	5,000–9,999 CNY	0.983 (0.753–1.283)	0.900	–	–
	≥10,000 CNY	0.759 (0.577–1.024)	0.073	–	–
	Refused to disclose	0.706 (0.469–1.062)	0.095	–	–
Occupation	Other healthcare workers	Reference	–	–	–
	Physician	1.031 (0.842–1.262)	0.767	–	–
	Nurse	0.894 (0.735–1.089)	0.267	–	–
History of influenza-like illness symptoms in the past year	No or unsure	Reference	–	–	
	Yes	1.141 (0.974–1.336)	0.102	–	
Participation in influenza vaccine-related training/publicity	No	Reference	–	Reference	–
	Yes	3.272(2.467–4.340)	<0.001	2.450(1.812–3.311)	<0.001
Institution provides influenza vaccination services	No	Reference	–	Reference	–
	Yes	2.210 (1.753–2.786)	<0.001	1.429 (1.065–1.918)	0.017
Free influenza vaccination policy	No	Reference	–	Reference	–
	Yes	3.914 (2.822–5.428)	<0.001	2.890 (1.922–4.346)	<0.001
Institutional attitude toward influenza vaccination among healthcare workers	Neither requiring nor encouraging	Reference	-	Reference	-
	Requiring vaccination	11.745 (7.376–18.701)	<0.001	6.807 (4.184–11.073)	<0.001
	Encouraging without requiring	3.305 (2.105–5.189)	<0.001	2.058 (1.285–3.297)	0.003
Knowledge score		1.254 (1.199–1.312)	<0.001	1.220 (1.161–1.282)	<0.001

aOR, adjusted odds ratio; CI, confidence interval.

Additional analysis showed that, compared with the subgroup without a free vaccination policy and with neither requiring nor encouraging vaccination, significantly higher odds of influenza vaccination uptake were observed among healthcare workers in the subgroups of free policy plus requiring vaccination, free policy plus encouraging vaccination without requiring it, and no free policy plus requiring vaccination (Supplementary Table S1).

### 3.3 Factors associated with intention to receive influenza vaccination during the 2026–2027 season

The univariate and multivariable logistic regression results for intention to receive influenza vaccination during the 2026–2027 season are presented in Table 3. In the multivariable model, years of professional practice, history of influenza-like illness symptoms in the past year, institutional attitude toward influenza vaccination, knowledge score, and influenza vaccination uptake during the 2025–2026 season were independently associated with future vaccination intention. Higher knowledge score, a supportive institutional attitude, and prior influenza vaccination uptake were positively associated with intention to receive influenza vaccination in the next season. Healthcare workers who had received influenza vaccination during the 2025–2026 season were substantially more likely to report intention to be vaccinated in the 2026–2027 season (aOR = 7.774, 95% CI: 6.401–9.440, *p* < 0.001). Multicollinearity diagnostics showed no significant collinearity among variables included in the multivariable model (all VIF values <3). The Hosmer–Lemeshow test suggested good model fit (*p* = 0.440), and the model demonstrated acceptable discriminative ability (AUC = 0.874).

**Table 3:** Univariate and multivariable logistic regression analyses of factors associated with intention to receive influenza vaccination during the 2026–2027 season.

Variable	Category/unit	Univariate OR (95% CI)	*p* value	Multivariable aOR (95% CI)	*p* value
Sex	Male	Reference	–	Reference	–
	Female	1.037 (0.893–1.204)	0.638	–	–
Age group, years	18–29	Reference	–	Reference	–
	30–39	0.894 (0.756–1.056)	0.188	–	–
	40–49	0.895 (0.748–1.071)	0.227	–	–
	≥50	0.967 (0.783–1.194)	0.753	–	–
Educational level	Secondary school or below	Reference	–	–	–
	Junior college	0.724 (0.436–1.201)	0.211	–	–
	Bachelor’s degree	0.670 (0.411–1.093)	0.109	–	–
	Master’s degree or above	0.692 (0.419–1.142)	0.150	–	–
Years of professional practice	≥20	Reference	–	Reference	–
	<5	1.223 (1.017–1.471)	0.033	1.349 (1.085–1.677)	0.007
	5–9	0.912 (0.753–1.104)	0.346	0.993 (0.795–1.242)	0.953
	10–19	1.021 (0.875–1.191)	0.791	1.189 (0.993–1.425)	0.060
Type of employing institution	Tertiary medical institution	Reference	–	Reference	–
	Secondary medical institution	1.251 (0.978–1.601)	0.075	–	–
	Primary medical institution	1.726 (1.478–2.016)	<0.001	–	–
	Ungraded medical institution	1.095 (0.828–1.449)	0.524	–	–
Occupational post	Clinical staff	Reference	–	Reference	–
	Administrative staff	1.052 (0.813–1.361)	0.700	–	–
	Public health staff	2.016 (1.543–2.634)	<0.001	–	–
	Medical technical staff	0.952 (0.831–1.090)	0.477	–	–
Professional title	No professional title	Reference	–	–	–
	Junior title	0.926 (0.706–1.214)	0.577	–	–
	Intermediate title	0.882 (0.674–1.154)	0.361	–	–
	Senior title	0.903 (0.666–1.225)	0.513	–	–
Monthly income	<5,000 CNY	Reference	–	–	–
	5,000–9,999 CNY	1.014 (0.807–1.274)	0.906	–	–
	≥10,000 CNY	0.929 (0.730–1.184)	0.552	–	–
	Refused to disclose	0.554 (0.393–0.781)	0.001	–	–
Occupation	Other healthcare workers	Reference	–	–	–
	Physician	1.165 (0.981–1.384)	0.082	–	–
	Nurse	1.120 (0.949–1.322)	0.179	–	–
History of influenza-like illness symptoms in the past year	No or unsure	Reference	–	Reference	–
	Yes	1.209 (1.058–1.382)	0.005	1.198 (1.025–1.401)	0.023
Participation in influenza vaccine-related training/publicity	No	Reference	–	Reference	–
	Yes	1.905(1.590–2.282)	<0.001	–	–
Institution provides influenza vaccination services	No	Reference	–	Reference	–
	Yes	1.411 (1.197–1.662)	<0.001	–	–
Free influenza vaccination policy	No	Reference	–	Reference	–
	Yes	1.982 (1.631–2.409)	<0.001	–	–
Institutional attitude toward influenza vaccination among healthcare workers	Neither requiring nor encouraging	Reference	–	Reference	–
	Requiring vaccination	8.528 (6.249–11.639)	<0.001	4.375 (3.100–6.174)	<0.001
	Encouraging without requiring	2.689 (2.034–3.555)	<0.001	1.713 (1.261–2.327)	0.001
Knowledge score		1.473 (1.415–1.533)	<0.001	1.461 (1.397–1.528)	<0.001
Influenza vaccination uptake during the 2025–2026 season	No	Reference	–	Reference	–
	Yes	10.034 (8.338–12.074)	<0.001	7.774 (6.401–9.440)	<0.001

aOR, adjusted odds ratio; CI, confidence interval.

Further analyses based on the BeSD framework suggested that, among the 1,852 healthcare workers willing to receive influenza vaccination during the 2026–2027 season, the thoughts and feelings domain appeared to play the most prominent role. The leading drivers were concern about transmitting influenza to family members or colleagues (88.3% agreed), belief in the effectiveness of influenza vaccination (87.9% agreed), and concern about the impact of influenza infection on work and daily life (86.1% agreed) (Supplementary Figure S1). Among the 2,386 healthcare workers unwilling or undecided about future influenza vaccination, the leading barriers were concern about adverse reactions (33.0% agreed), perceived good health and no need for vaccination (33.0% agreed), and the belief that influenza is mild and has little impact (29.6% agreed) (Supplementary Figure S2).

### 3.4 Factors associated with willingness to recommend influenza vaccination to patients

The univariate and multivariable logistic regression results for willingness to recommend influenza vaccination to patients are presented in Table 4. In the multivariable model, type of employing institution, participation in influenza vaccine-related training/publicity, free vaccination policy, institutional attitude toward influenza vaccination, knowledge score, influenza vaccination uptake during the 2025–2026 season, and intention to receive influenza vaccination during the 2026–2027 season were independently associated with willingness to recommend influenza vaccination to patients. In particular, a supportive institutional attitude, higher knowledge score, prior influenza vaccination uptake, and future vaccination intention were positively associated with recommendation willingness. Healthcare workers who intended to receive influenza vaccination during the 2026–2027 season were substantially more likely to recommend influenza vaccination to patients (aOR = 6.349, 95% CI: 4.766–8.457, *p* < 0.001). No evidence of problematic multicollinearity was observed among variables in the multivariable model (all VIF values <3). The model calibration was satisfactory based on the Hosmer–Lemeshow test (*p* = 0.332), and the predictive performance was acceptable (AUC = 0.846).

**Table 4:** Univariate and multivariable logistic regression analyses of factors associated with willingness to recommend influenza vaccination to patients.

Variable	Category/unit	Univariate OR (95% CI)	P value	Multivariable aOR (95% CI)	P value
Sex	Male	Reference	–	Reference	–
	Female	1.042 (0.861–1.261)	0.674	–	–
Age group, years	18–29	Reference	–	Reference	–
	30–39	1.182 (0.954–1.465)	0.126	–	–
	40–49	1.031 (0.821–1.294)	0.793	–	–
	≥50	1.052 (0.804–1.377)	0.711	–	–
Educational level	Secondary school or below	Reference	–	–	–
	Junior college	0.868 (0.452–1.667)	0.671	–	–
	Bachelor’s degree	1.029 (0.546–1.938)	0.931	–	–
	Master’s degree or above	1.088 (0.568–2.082)	0.800	–	–
Years of professional practice	≥20	Reference	–	–	–
	<5	1.062 (0.835–1.352)	0.623	–	–
	5–9	0.950 (0.746–1.210)	0.677	–	–
	10–19	1.023 (0.839–1.248)	0.821	–	–
Type of employing institution	Tertiary medical institution	Reference	–	Reference	–
	Secondary medical institution	0.895 (0.662–1.211)	0.473	1.038 (0.728–1.479)	0.837
	Primary medical institution	1.849 (1.466–2.334)	<0.001	1.524 (1.171–1.983)	0.002
	Ungraded medical institution	0.832 (0.596–1.161)	0.279	1.357 (0.887–2.078)	0.160
Occupational post	Clinical staff	Reference	–	Reference	–
	Administrative staff	0.922 (0.668–1.274)	0.623	–	–
	Public health staff	2.091 (1.356–3.226)	0.001	–	–
	Medical technical staff	0.982 (0.826–1.167)	0.836	–	–
Professional title	No professional title	Reference	–	–	–
	Junior title	1.215 (0.870–1.696)	0.254	–	–
	Intermediate title	1.268 (0.910–1.767)	0.160	–	–
	Senior title	1.106 (0.760–1.610)	0.599	–	–
Monthly income	<5,000 CNY	Reference	–	–	–
	5,000–9,999 CNY	1.534 (1.166–2.020)	0.002	–	–
	≥10,000 CNY	1.277 (0.955–1.707)	0.099	–	–
	Refused to disclose	0.979 (0.664–1.443)	0.915	–	–
Occupation	Other healthcare workers	Reference	–	–	–
	Physician	1.321 (1.064–1.640)	0.012	–	–
	Nurse	1.199 (0.977–1.472)	0.082	–	–
History of influenza-like illness symptoms in the past year	No or unsure	Reference	–	–	–
	Yes	1.053 (0.885–1.253)	0.562	–	–
Participation in influenza vaccine-related training/publicity	No	Reference	–	Reference	–
	Yes	2.319(1.915–2.807)	<0.001	1.375(1.096–1.725)	0.006
Institution provides influenza vaccination services	No	Reference	–	Reference	–
	Yes	1.613 (1.333–1.951)	<0.001	–	–
Free influenza vaccination policy	No	Reference	–	Reference	–
	Yes	2.115 (1.724–2.594)	<0.001	1.323 (1.023–1.711)	0.033
Institutional attitude toward influenza vaccination among healthcare workers	Neither requiring nor encouraging	Reference	–	Reference	–
	Requiring vaccination	6.943 (4.979–9.682)	<0.001	2.238 (1.516–3.303)	<0.001
	Encouraging without requiring	3.249 (2.561–4.123)	<0.001	1.876 (1.419–2.480)	<0.001
Knowledge score		1.632 (1.560–1.708)	<0.001	1.438 (1.370–1.509)	<0.001
Influenza vaccination uptake during the 2025–2026 season	No	Reference	–	Reference	–
	Yes	5.511 (4.040–7.519)	<0.001	1.630 (1.145–2.319)	0.007
Intention to receive influenza vaccination during the 2026–2027 season	No	Reference	–	Reference	–
	Yes	11.814 (9.052–15.417)	<0.001	6.349 (4.766–8.457)	<0.001

aOR, adjusted odds ratio; CI, confidence interval.

Among the 764 healthcare workers unwilling to recommend influenza vaccination to patients, the leading barriers were concern about adverse reactions in patients (78.3%), concern about perceived commercial motives (59.9%), and concern about patient dissatisfaction or disputes (49.5%) (Supplementary Figure S3). In addition, a survey of all 4,238 healthcare workers regarding the implementation of an adult vaccine prescription strategy showed that the main perceived challenges were lack of unified and well-established policies (66.8%), insufficient vaccination staff and space (53.2%), and lack of incentive mechanisms (49.9%) (Supplementary Figure S4).

### 3.5 SEM-based path analysis

**Figure 1:**
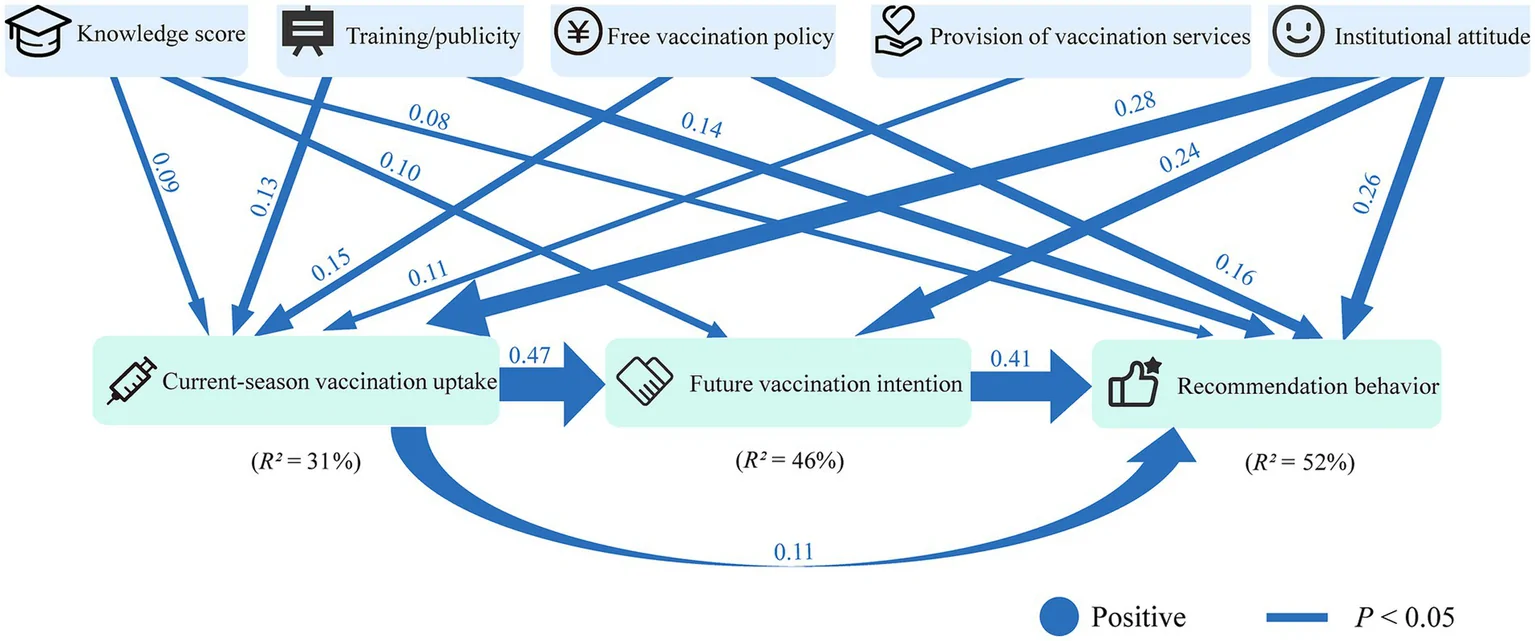
SEM-based path analysis model illustrating the associations among influenza vaccine-related knowledge, current-season vaccination uptake, future vaccination intention, and recommendation behavior among healthcare workers. Arrows represent hypothesized statistical associations rather than causal relationships.

The SEM-based observed-variable path analysis model showed a good fit to the data (CFI = 0.957, TLI = 0.941, RMSEA = 0.041, and SRMR = 0.036). Given that the model included categorical observed outcomes, parameter estimation was performed using the WLSMV estimator. Because the model consisted exclusively of observed variables, no latent measurement model was specified and CFA-derived parameters were not estimated. The path diagram and the direct, indirect, and total effects are presented in Figure 1 and Table 5, respectively.

**Table 5:** Direct, indirect, and total effects in the SEM-based path analysis model.

Pathway		Standardized effect (*β*)	SE	95% CI	*p* value
Direct effects	Knowledge score → Current-season vaccination uptake	0.09	0.01	0.06, 0.13	<0.001
	Training/publicity → Current-season vaccination uptake	0.13	0.02	0.08, 0.17	<0.001
	Free vaccination policy → Current-season vaccination uptake	0.15	0.02	0.10, 0.19	<0.001
	Provision of vaccination services → Current-season vaccination uptake	0.11	0.04	0.03, 0.19	0.008
	Institutional attitude → Current-season vaccination uptake	0.28	0.03	0.21, 0.34	<0.001
	Knowledge score → Future vaccination intention	0.10	0.02	0.06, 0.13	<0.001
	Institutional attitude → Future vaccination intention	0.24	0.03	0.18, 0.29	<0.001
	Current-season vaccination uptake → Future vaccination intention	0.47	0.04	0.39, 0.54	<0.001
	Knowledge score → Recommendation behavior	0.08	0.02	0.05, 0.14	<0.001
	Training/publicity → Recommendation behavior	0.14	0.03	0.02, 0.18	<0.001
	Free vaccination policy → Recommendation behavior	0.16	0.02	0.11, 0.18	<0.001
	Institutional attitude → Recommendation behavior	0.26	0.03	0.19, 0.33	0.001
	Current-season vaccination uptake → Recommendation behavior	0.11	0.04	0.03, 0.18	0.004
	Future vaccination intention → Recommendation behavior	0.41	0.03	0.34, 0.47	<0.001
Indirect effects	Knowledge score → Current-season vaccination uptake → Future vaccination intention	0.11	0.02	0.06, 0.15	<0.001
	Knowledge score → Current-season vaccination uptake → Future vaccination intention → Recommendation behavior	0.06	0.01	0.02, 0.13	<0.001
	Current-season vaccination uptake → Future vaccination intention → Recommendation behavior	0.27	0.04	0.12, 0.24	<0.001
Total effects	Knowledge score → Future vaccination intention	0.21	0.03	0.15, 0.26	<0.001
	Knowledge score → Recommendation behavior	0.14	0.03	0.08, 0.19	<0.001
	Current-season vaccination uptake → Recommendation behavior	0.38	0.04	0.29, 0.46	<0.001

SE, standard error; CI, confidence interval.

Path analysis showed that knowledge score was positively associated with influenza vaccination uptake during the current influenza season (*β* = 0.09, *p* < 0.001). Training/publicity (*β* = 0.13, *p* < 0.001), free vaccination policy (*β* = 0.15, *p* < 0.001), provision of vaccination services (*β* = 0.11, *p* = 0.008), and institutional attitude toward influenza vaccination (*β* = 0.28, *p* < 0.001) were also positively associated with current-season vaccination uptake.

For intention to receive influenza vaccination in the next influenza season, knowledge score (*β* = 0.10, *p* < 0.001), institutional attitude toward influenza vaccination (*β* = 0.24, *p* < 0.001), and current-season vaccination uptake (*β* = 0.47, *p* < 0.001) all showed significant positive effects, with current-season vaccination uptake showing the strongest association.

For willingness to recommend influenza vaccination to patients, future vaccination intention had the strongest direct positive effect (*β* = 0.41, *p* < 0.001). Current-season vaccination uptake (*β* = 0.11, *p* = 0.004) and knowledge score (*β* = 0.08, *p* < 0.001) were also directly and positively associated with recommendation behavior. In addition, knowledge score, training/publicity, free vaccination policy, and institutional attitude toward influenza vaccination were also positively associated with recommendation behavior.

Mediation analysis based on the SEM framework showed that knowledge score was statistically associated with future vaccination intention through current-season vaccination uptake (*β* = 0.11, *p* < 0.001), and a significant chain-mediated indirect effect on recommendation behavior through current-season vaccination uptake and future vaccination intention (*β* = 0.06, *p* < 0.001). Current-season vaccination uptake also had a significant indirect effect on recommendation behavior through future vaccination intention (*β* = 0.27, *p* < 0.001). The model explained 31% of the variance in current-season vaccination uptake, 46% of the variance in future vaccination intention, and 52% of the variance in recommendation behavior.

A Harman’s single-factor test was conducted to evaluate the potential presence of common method bias. The exploratory factor analysis identified multiple factors, and the first factor accounted for 31.3% of the total variance, which was below the recommended threshold of 50%. These findings suggested that common method bias was unlikely to substantially affect the observed associations.

## 4 Discussion

In this cross-sectional survey of 4,238 healthcare workers in Shijingshan District, Beijing, we found that influenza vaccination uptake during the 2025–2026 season was relatively low, whereas willingness to recommend influenza vaccination to patients was comparatively high. Intention to receive influenza vaccination in the next influenza season remained at a moderate level. More importantly, our findings suggest that influenza vaccination-related behaviors among healthcare workers are shaped by a combination of individual knowledge, prior vaccination behavior, and institutional support. The SEM-based observed-variable path model further revealed a clear behavioral pathway linking knowledge score, current-season vaccination uptake, future vaccination intention, and recommendation behavior.

First, influenza vaccination uptake during the current season was independently associated with knowledge score, training/publicity, free vaccination policy, provision of vaccination services, and institutional attitude toward influenza vaccination. These findings indicate that actual vaccination behavior among healthcare workers is influenced not only by individual cognition, but also by the practical and organizational environment in which vaccination decisions are made (11). In particular, free vaccination policy and institutional encouragement or requirements appear to play an important facilitating role. This suggests that improving access and reducing structural barriers may be as important as enhancing knowledge in promoting vaccine uptake among healthcare workers (12).

In addition, compared with healthcare workers with ≥20 years of work experience, those with <5 years and 5–9 years of experience both showed higher influenza vaccination uptake. This finding suggests that relatively early- and mid-career healthcare workers tend to have better adherence to influenza vaccination, whereas long-tenured staff exhibit lower uptake (13). Several explanations may account for this pattern. First, healthcare workers with shorter and intermediate years of service are more likely to have recently received structured training and continuing education emphasizing infection prevention and control, which may enhance their compliance with vaccination recommendations (14). Second, this group may have a stronger perception of occupational exposure risk and a greater willingness to adopt preventive measures early in their careers. In contrast, more experienced healthcare workers may develop a sense of habituation or perceived invulnerability due to long-term clinical exposure, leading to risk underestimation and reduced motivation for annual influenza vaccination (15). Therefore, differentiated intervention strategies targeting healthcare workers with longer work experience may be necessary to improve overall vaccination coverage.

Second, intention to receive influenza vaccination in the next season was strongly associated with prior vaccination uptake, indicating substantial behavioral continuity. Healthcare workers who had been vaccinated during the 2025–2026 season were far more likely to express willingness to be vaccinated again in the following season. This finding suggests that one successful vaccination experience may reinforce future acceptance and help establish stable vaccination habits (16). In addition, knowledge score and institutional attitude were also positively associated with future vaccination intention, indicating that both cognitive understanding and workplace norms contribute to sustaining willingness over time (17).

Third, willingness to recommend influenza vaccination to patients was most strongly associated with future vaccination intention, followed by prior vaccination uptake and knowledge score. This pattern suggests that healthcare workers’ recommendation behavior is closely related to their own vaccine-related beliefs and practices. In other words, healthcare workers who are more willing to vaccinate themselves are also more likely to recommend vaccination to patients (18). This finding has important practical implications, as healthcare workers serve not only as potential vaccine recipients but also as key communicators and advocates for adult vaccination (16).

The supplementary BeSD-based analyses provided additional insight into the psychological and social mechanisms underlying future vaccination intention. Among healthcare workers willing to receive influenza vaccination, the most prominent drivers were concern about transmitting influenza to family members or colleagues, belief in vaccine effectiveness, and concern about the impact of influenza infection on work and daily life. These results suggest that both self-protection and responsibility toward others are important motivations for vaccine acceptance. By contrast, among those unwilling or undecided, the leading barriers included concern about adverse reactions, perceived good health and no need for vaccination, and the belief that influenza is mild and has little impact (3). Together, these findings suggest that future interventions should not only provide knowledge, but also directly address risk perception, safety concerns, and misconceptions regarding influenza severity.

An important finding of this study was the substantial discrepancy between personal influenza vaccination uptake and willingness to recommend vaccination to patients. Although only 22.1% of healthcare workers had received influenza vaccination during the 2025–2026 season, 82.0% reported willingness to recommend influenza vaccination to patients. This discrepancy suggests that personal vaccination behavior and professional recommendation behavior should not be considered equivalent. Personal vaccination decisions may be more strongly influenced by individual risk perception, perceived need, concerns about adverse reactions, and convenience. Consistent with this interpretation, among healthcare workers who were unwilling or undecided about receiving influenza vaccination in the following season, the leading barriers included concerns about adverse reactions, the perception that they were healthy and did not need vaccination, and the belief that influenza was mild and had limited impact. In contrast, recommendation behavior may additionally reflect professional responsibility, knowledge of patients’ risk factors, and the role of healthcare workers in preventive health education. Healthcare workers may therefore recognize the benefits of influenza vaccination for patients, particularly those at increased risk of influenza-related complications, even when they do not perceive the same level of need for themselves. The relatively strong association between future vaccination intention and recommendation behavior in our study further suggests that personal vaccine attitudes remain relevant, but recommendation behavior is not simply a direct reflection of personal vaccination uptake. The discrepancy observed in this study may therefore reflect the coexistence of individual-level barriers to personal vaccination and professional or patient-centered motivation to promote vaccination.

Similarly, among healthcare workers unwilling to recommend influenza vaccination to patients, the most commonly reported barriers were concern about adverse reactions in patients, concern about perceived commercial motives, and concern about patient dissatisfaction or disputes. These findings indicate that recommendation behavior is affected not only by scientific knowledge, but also by concerns about communication risks and the doctor–patient relationship (1). Therefore, improving recommendation behavior may require more than educational interventions alone; it may also require institutional endorsement, communication training, and clearer professional norms regarding adult vaccination recommendation (9).

In addition, the survey on barriers to implementing an adult vaccine prescription strategy showed that healthcare workers were particularly concerned about the lack of unified policies, insufficient vaccination staff and space, and lack of incentive mechanisms. These findings suggest that promoting vaccine prescription strategies in clinical settings requires system-level support. Policy coordination, workflow integration, human resource allocation, and incentive mechanisms may all be essential for successful implementation. Taken together, our findings support a multi-level intervention approach that combines knowledge promotion, institutional support, service accessibility, and policy reinforcement (7).

Occupational role may also influence healthcare workers’ influenza vaccination-related behaviors and recommendation practices. In the present study, occupation was considered in the multivariable analyses of all three primary outcomes. Although some occupational differences were observed in the unadjusted analyses, neither physician nor nurse status remained significantly associated with vaccination uptake, future vaccination intention, or willingness to recommend influenza vaccination after adjustment for other relevant factors. This suggests that the observed differences may be partly explained by individual and organizational factors rather than occupation itself. In the Chinese healthcare setting, both physicians and nurses may participate in vaccine-related health education, information provision, and vaccination recommendations according to their clinical roles and institutional practices. However, the final assessment of vaccination eligibility and the administration of vaccines are generally undertaken by designated preventive healthcare or immunization service departments. Therefore, recommendation by physicians and nurses should be understood primarily as professional health education and guidance that facilitates appropriate vaccination, rather than as formal vaccine prescription or administration. This distinction is important when interpreting recommendation behavior in the Chinese healthcare context.

The SEM-based path analysis further provided insights into the potential associative relationships among influenza vaccine-related knowledge, vaccination uptake, vaccination intention, and recommendation behavior. From a behavioral perspective, these findings may reflect a sequential process in which knowledge acquisition represents an initial cognitive stage, vaccination uptake represents the transition from intention to action, and recommendation behavior represents the extension of personal behavior into professional practice (3). Knowledge alone may be insufficient to produce behavioral change unless it is translated into actual vaccination experiences. Once healthcare workers receive influenza vaccination themselves, positive vaccination experiences may enhance confidence in vaccine effectiveness and safety, thereby reinforcing future vaccination intention and increasing their willingness to recommend vaccination to patients (2). Knowledge score was significantly associated with current-season vaccination uptake, future vaccination intention, and recommendation behavior, and showed indirect associations with intention and recommendation behavior through vaccination uptake (19). Current-season vaccination uptake also showed an indirect association with recommendation behavior through future vaccination intention (20). Although the proposed behavioral pathway from knowledge to vaccination uptake, intention, and recommendation behavior is theoretically plausible, these findings should be interpreted as associative patterns rather than evidence of causal mechanisms, given the cross-sectional design of this study. Knowledge may represent an important factor associated with vaccine-related behaviors, while the temporal sequence and causal relationships among these variables require further investigation using longitudinal designs (7). At the same time, institutional factors such as training/publicity, free vaccination policy, provision of vaccination services, and institutional attitude showed meaningful direct effects within the model, highlighting the importance of supportive organizational contexts (4). These findings highlight that healthcare workers’ vaccination behaviors are not determined solely by individual cognition but are embedded within an organizational context. Supportive institutional environments may function through multiple behavioral pathways, including reducing practical barriers, increasing perceived social norms, and signaling organizational endorsement of vaccination (21). Therefore, interventions focusing only on knowledge improvement may have limited effects if healthcare workers continue to face accessibility constraints or lack institutional encouragement (2).

Overall, these findings suggest that improving influenza vaccination-related behaviors among healthcare workers requires coordinated efforts at multiple levels. Educational interventions may improve vaccine-related knowledge and correct misconceptions, while institutional and policy measures may reduce practical barriers and reinforce supportive norms. In addition, promoting healthcare workers’ own vaccination uptake may have broader downstream effects on future vaccination intention and recommendation behavior, thereby potentially contributing to wider public acceptance of influenza vaccination. Our findings extend previous research by suggesting that influenza vaccination promotion among healthcare workers should not be viewed as a single behavioral outcome but rather as a dynamic behavioral pathway. Improving knowledge may initiate behavioral change; however, converting knowledge into personal vaccination practice and establishing supportive organizational environments may be critical steps for achieving sustained vaccination behaviors and enhancing healthcare workers’ role as vaccine advocates.

### 4.1 Strengths

This study has several strengths. First, it was based on a relatively large sample of healthcare workers drawn from different types of medical institutions in Shijingshan District, which improves the representativeness of the findings within the local healthcare setting. Second, the study examined multiple influenza vaccination-related outcomes simultaneously, including current-season vaccination uptake, future vaccination intention, and willingness to recommend influenza vaccination to patients. This allowed us to capture different stages of vaccine-related attitudes and behaviors among healthcare workers. Third, in addition to conventional logistic regression analyses, this study incorporated supplementary analyses based on the BeSD framework and explored barriers to recommendation behavior and implementation of an adult vaccine prescription strategy. These analyses provided a more comprehensive understanding of the cognitive, social, and organizational factors influencing influenza vaccination-related behaviors. Finally, the use of SEM-based observed-variable path model enabled us to further clarify the behavioral pathway relationships among knowledge score, vaccination uptake, vaccination intention, and recommendation behavior, thereby strengthening the interpretation of the observed associations.

### 4.2 Limitations

Several limitations should be acknowledged.

First, this was a cross-sectional study, and therefore causal relationships cannot be established. Although SEM was applied based on a theoretically informed framework and demonstrated good model fit, the estimated behavioral pathways represent statistical associations rather than confirmed causal mechanisms (22). In particular, the hypothesized sequence from knowledge to vaccination uptake, vaccination intention, and recommendation behavior should be interpreted cautiously because the temporal order of these variables cannot be determined from cross-sectional data. Future longitudinal studies are needed to verify the directionality and causal relationships among these factors. Another potential limitation is the possible influence of the preceding 2024–2025 influenza season on vaccination-related behaviors during the 2025–2026 season. Experiences of influenza infection and perceptions of influenza activity in the previous season may influence subsequent risk perception, vaccination decisions, and willingness to recommend vaccination. In the present study, a history of influenza-like illness symptoms during the preceding year was collected and incorporated into the multivariable analysis of future vaccination intention (23). However, we did not specifically assess participants’ perceptions of the severity of the 2024–2025 influenza season, and individual-level vaccination decisions could not be directly linked to objective measures of influenza activity during that season. Therefore, a potential temporal influence of the preceding influenza season cannot be completely excluded. Future longitudinal studies integrating individual vaccination histories, perceptions of influenza risk, and objective influenza surveillance indicators across multiple seasons are warranted to further clarify these relationships. In addition, the SEM analysis in this study was conducted using directly measured observed variables rather than latent constructs. Therefore, measurement model assessments, including factor loadings, composite reliability, and average variance extracted, were not performed. Future studies incorporating validated multi-item measurement scales may further apply latent variable SEM approaches to explore the underlying mechanisms of influenza vaccination-related behaviors.

Second, the data were collected through self-reported questionnaires, and therefore vaccination uptake, future vaccination intention, and recommendation behavior may be subject to recall bias and social desirability bias. Although anonymous data collection and multiple quality-control procedures were applied to improve data reliability, objective verification using vaccination records or institutional immunization databases was not available in this study. Future studies combining questionnaire-based assessments with objectively recorded vaccination information may further improve the accuracy and validity of findings (24).

Third, this study was conducted among healthcare workers in medical institutions within a single district of Beijing, which may limit the generalizability of the findings to other regions or healthcare systems. Although stratified two-stage sampling was used and participants were recruited from healthcare institutions with different levels and occupational categories, differences in vaccination policies, healthcare resources, and organizational cultures across regions may influence influenza vaccination-related behaviors (25). In addition, healthcare workers were clustered within medical institutions and may therefore have shared similar organizational environments and vaccination policies. Although institutional characteristics, including free vaccination policies, provision of vaccination services, and institutional attitudes toward influenza vaccination, were incorporated as covariates in the present analyses, the current analytical framework did not explicitly model the hierarchical structure of healthcare workers nested within institutions. Therefore, potential residual institution-level clustering effects cannot be completely excluded. Future multicenter studies involving a larger number of healthcare institutions and more comprehensive institution-level variables could apply multilevel modeling to distinguish individual-level effects from institution-level contextual effects and to further evaluate how organizational vaccination policies influence healthcare workers’ vaccination-related behaviors.

Fourth, although a range of sociodemographic, occupational, knowledge-related, and institutional factors were included in the analyses, some potentially important determinants of influenza vaccination behavior were not assessed. These included vaccine confidence, previous adverse vaccine experiences, chronic health conditions, trust in health authorities, and more detailed aspects of organizational culture. The absence of these variables may have resulted in residual confounding and may have influenced the observed associations. Future studies incorporating broader behavioral, psychological, and organizational factors are needed to provide a more comprehensive understanding of influenza vaccination-related behaviors among healthcare workers.

Finally, although the questionnaire was adapted from a previously validated instrument and demonstrated satisfactory reliability and validity in the pilot study of the present research, several items were added or adjusted to better reflect the local healthcare context. Therefore, differences in questionnaire content and contextual characteristics should be considered when comparing our findings with those from other populations or healthcare settings.

## 5 Conclusion

In conclusion, influenza vaccination-related behaviors among healthcare workers were influenced by a combination of knowledge, prior vaccination experience, and institutional support. Current-season vaccination uptake, future vaccination intention, and willingness to recommend influenza vaccination to patients were closely interconnected. Knowledge score played both direct and indirect roles in shaping these vaccine-related behaviors, while training/publicity, free vaccination policy, provision of vaccination services, and institutional attitude toward influenza vaccination also contributed meaningfully.

These findings suggest that efforts to improve influenza vaccination among healthcare workers should go beyond knowledge promotion alone. Multi-level strategies, including educational interventions, improved service accessibility, supportive institutional policies, and clearer organizational norms for vaccination and vaccine recommendation, may be needed to enhance both personal vaccination uptake and professional recommendation behavior. Such efforts may ultimately contribute to broader acceptance and promotion of influenza vaccination in the general population.

## Data Availability

The raw data supporting the conclusions of this article will be made available by the authors, without undue reservation.
