# Supplementary material for: Behavioral pathways linking influenza vaccine knowledge, uptake, vaccination intention, and recommendation behavior among healthcare workers: a cross-sectional study in Shijingshan District, Beijing
Source: Front Public Health. 2026 Sep 9;14:1886295. doi: 10.3389/fpubh.2026.1886295 (PMC13597684; doi:10.3389/fpubh.2026.1886295)
Supplement: Supplementary file 1 [file Supplementary_file_1.DOCX]

Supplementary Table S1 Association of different combinations of free vaccination policy and institutional attitude with influenza vaccination uptake during the 2025–2026 season

| Free vaccination policy | Institutional attitude | No. of participants (n=4238) | No. vaccinated (n=936) | Vaccination rate, % | aOR (95% CI) | *P* value |
| --- | --- | --- | --- | --- | --- | --- |
| Free policy | requiring vaccination | 704 | 323 | 45.9 | 14.366 (5.976–34.537) | ＜0.001 |
|  | encouraging without requiring | 2796 | 557 | 19.9 | 4.328 (1.815–10.321) | 0.001 |
|  | neither requiring nor encouraging | 195 | 15 | 7.7 | 1.874 (0.680–5.161) | 0.225 |
| No free policy | requiring vaccination | 39 | 8 | 20.5 | 4.300 (1.348–13.712) | 0.014 |
|  | encouraging without requiring | 371 | 27 | 7.3 | 1.450 (0.576–3.649) | 0.430 |
|  | neither requiring nor encouraging | 133 | 6 | 4.5 | Reference | - |

**Note:** Adjusted for years of professional practice, type of employing institution, occupational post, participation in influenza vaccine-related training/publicity, provision of influenza vaccination services, and knowledge score. aOR, adjusted odds ratio; CI, confidence interval.
